# MALDI TOF Mass Spectrometry Imaging of Blood Smear: Method Development and Evaluation

**DOI:** 10.3390/ijms22020585

**Published:** 2021-01-08

**Authors:** Željko Debeljak, Ann-Christin Niehoff, Ana Bandjak, Dario Mandić, Bojana Bošnjak, Marija Heffer, Stefan Mrđenović, Ivana Marković, Milorad Zjalić, Vatroslav Šerić

**Affiliations:** 1 Clinical Institute of Laboratory Diagnostics, Osijek University Hospital, J. Huttlera 4, 31 000 Osijek, Croatia; zeljko.debeljak@gmail.com (Ž.D.); ana.bandjak@gmail.com (A.B.); dario.mandic@gmail.com (D.M.); seric.vatroslav@kbo.hr (V.Š.); 2 Department of Pharmacology, Faculty of Medicine, JJ Strossmayer University of Osijek, J. Huttlera 4, 31 000 Osijek, Croatia; 3 European Innovation Center, Shimadzu Europa GmbH, Albert-Hahn-Straße 6-10, 47269 Duisburg, Germany; acn@shimadzu.eu; 4 Department of Medical Chemistry, Biochemistry and Clinical Chemistry, Faculty of Medicine, JJ Strossmayer University of Osijek, J. Huttlera 4, 31 000 Osijek, Croatia; 5 Clinical Institute of Transfusion Medicine, Osijek University Hospital, J. Huttlera 4, 31 000 Osijek, Croatia; bosnjak.bojana@kbo.hr; 6 Department of Microbiology, Parasitology and Clinical Laboratory Diagnostics, Faculty of Medicine, JJ Strossmayer University of Osijek, J. Huttlera 4, 31 000 Osijek, Croatia; 7 Department of Medical Biology and Genetics, Faculty of Medicine, JJ Strossmayer University of Osijek, J. Huttlera 4, 31 000 Osijek, Croatia; mheffer@mefos.hr (M.H.); mzjalic@mefos.hr (M.Z.); 8 Department of Hematology, Internal Medicine Clinic, Osijek University Hospital, J. Huttlera 4, 31 000 Osijek, Croatia; stefan.mrdenovic@kbc.hr; 9 Department of Internal Medicine, Family Medicine and History of Medicine, Faculty of Medicine Osijek, JJ Strossmayer University of Osijek, J. Huttlera 4, 31 000 Osijek, Croatia

**Keywords:** MALDI TOF, mass spectrometry imaging, blood smear, method development

## Abstract

The aim of this study was to develop and evaluate matrix assisted LASER desorption ionization (MALDI) time-of-flight (TOF) mass spectrometry imaging (MSI) of blood smear. Integrated light microscope and MALDI IT-TOF mass spectrometer, together with a matrix sublimation device, were used for analysis of blood smears coming from healthy male donors. Different blood plasma removal, matrix deposition, and instrumental settings were evaluated using the negative and positive ionization modes while agreement between the light microscopy images and the lateral distributions of cellular marker compounds served as the MSI quality indicator. Red and white blood cells chemical composition was analyzed using the differential *m*/*z* expression. Five seconds of exposure to ethanol followed by the 5 min of 9-aminoacridine or α-cyano-4-hydroxycinnamic acid deposition, together with two sets of instrumental settings, were selected for the MALDI TOF MSI experiments. Application of the thin and transparent matrix layers assured good correspondence between the LASER footprints and the preselected regions of interest. Cellular marker *m*/*z* signals coincided well with the appropriate cells. A metabolite databases search using the differentially expressed *m*/*z* produced hits which were consistent with the respective cell types. This study sets the foundations for application of blood smear MALDI TOF MSI in clinical diagnostics and research.

## 1. Introduction

Red blood cells (RBC) circulate for approximately 4 months through the human vascular system [1]. In this way, cells come in contact with each organ and tissue, which affects RBC appearance and leaves a unique signature. Different mechanical obstacles that may exist in circulation cause visible deformation of the blood cells. Even blood plasma chemistry changes, caused by some liver or kidney diseases, lead to specific RBC membrane chemical changes that manifest themselves as deformations detectable by light microscopy [2]. Unfortunately, the light microscopy of native or even stained blood smears cannot detect chemical signatures carried by blood cells which are caused by other organ pathologies. Flow cytometry and immunohistochemical techniques may partially overcome this shortage, but these techniques are not suitable for simultaneous assessment of numerous metabolites and other small molecules [1]. On the other end of the analytical spectrum lies liquid chromatography coupled to tandem mass spectrometry (LC-MS/MS), which enables quantification of different endo- and xenobiotics in blood cell homogenates but cannot assign contents of different small molecules to individual cells [3,4]. Also, many sample-preparation steps needed for separation of blood cell fractions, cellular homogenization, and homogenate prefractionation may compromise the LC-MS/MS-based approach to average RBC or white blood cell (WBC) chemical content determination and make this approach unsuitable for routine diagnostics.

Light microscopy-based morphology analysis and blood cell differentiation using blood smear is the least invasive yet informative procedure that is widely used in different medical fields like hematology, immunology, infectology, oncology, blood transfusion, organ transplantation, etc. [1]. Adding information about the chemical composition of individual cells to the already available count and morphology of blood cells is expected to bring significant advancements to the respective medical fields [5,6]. Blood cells that were in contact with tissues affected by emerging pathological process are expected to be tagged by a chemical imprint of the process. For timely diagnosis, a fraction of the blood cells that are chemically tagged should be detected. Mass spectrometry imaging (MSI) combines the advantages of light microscopy and MS, making it suitable for assessment of the chemical composition of individual blood cells present in blood smear. Different MSI techniques may provide information about the chemical composition of tissues and organs [7,8]. However, a blood cell diameter of 5–25 μm represents an important constraint for the application of most available MSI technologies. Of the commercially available techniques that can be applied in routine diagnostic settings, only the secondary ion mass spectrometry (SIMS) and some matrix-assisted LASER desorption/ionization (MALDI) time-of-flight (TOF) MS instruments provide a lateral resolution of 10 µm or better, which is required for reliable MSI of such small objects [9,10,11,12,13]. When it comes to blood smear that contains dozens of different blood cells per 400× magnification field, besides the need for high lateral resolution, application of the integrated mass and light microscopy instruments seems mandatory: shifts of even 5 µm may lead to miss-assignment of a recorded mass spectrum to cells that surround the cell that have been shot by a LASER beam.

Thanks to plasma, blood cells are easily attached to different surfaces including indium tin oxide (ITO) slides. However, plasma that covers all cells contains many different small molecules such as lipids, which should be removed if the cellular content is to be analyzed [14]. The least sample-integrity-compromising way to remove the plasma components is to immerse the ITO slide covered by the blood smear in a polar solvent such as ethanol (EtOH), keeping in mind the possibility of unwanted cell lysis and lateral diffusion. Besides the removal of interfering plasma components, ion suppression is also reduced this way [15]. To achieve the highest possible signal intensities coming from the small objects using MALDI TOF MSI, the prepared blood-smeared ITO slide should be covered by the appropriate matrix. The matrix crystals should be as small as possible to match the cells’ diameter: this can be achieved by sublimation [16]. To eliminate the possibility of ITO slide shifts, light microscopy images of blood smear should be recorded after the matrix has been applied: this way, MSI and light microscopy images are recorded without the need for any ITO slide movements. For that purpose, the matrix layer should be thin and transparent, which also makes the matrix deposition by sublimation a method of choice. The blood cell size also dictates the LASER setting selection. Obtaining a small LASER footprint while keeping a reasonable *m*/*z* signal intensity might be challenging [17,18]. To meet the requirements for a small LASER spot footprint, low LASER energies and a low number of shots per pixel should be used. This way, the mixing of spectra coming from neighboring cells is minimized.

The aim of this study was to develop and evaluate the MALDI TOF MSI of blood smear. To the best of our knowledge, this is the first attempt to achieve this goal. The approach described in the preceding text was applied, and correspondences between the cellular distribution obtained using light microscopy; the recorded lateral distribution of cellular markers such as heme, adenosine triphosphate (ATP), and inosine monophosphate (IMP); and the corresponding *m*/*z* signal intensities were used as the MALDI TOF MSI quality indicators. Differentially expressed *m*/*z* signals coming from RBC and WBC recorded using optimal sample preparation and instrumental conditions were analyzed.

## 2. Results

### 2.1. Method Development

**Figure 1:**
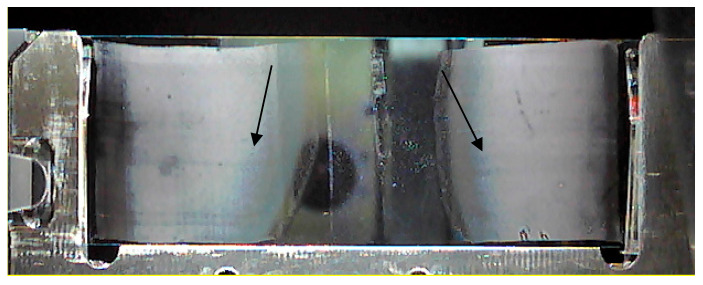
Two-microliter blood drops smeared on two ends of the same indium tin oxide (ITO) slide, with the arrows pointing to optimal cell dispersion zones: cells lying close to each other disable unequivocal mass spectrometry imaging (MSI) analysis, while too much dispersion of cells yields too few cells.

A macroscopic image of a holder containing an ITO slide covered by 2 blood smears is given in Figure 1. Due to optimal cell dispersion, a 2-µL blood drop volume was chosen.

**Figure 2:**
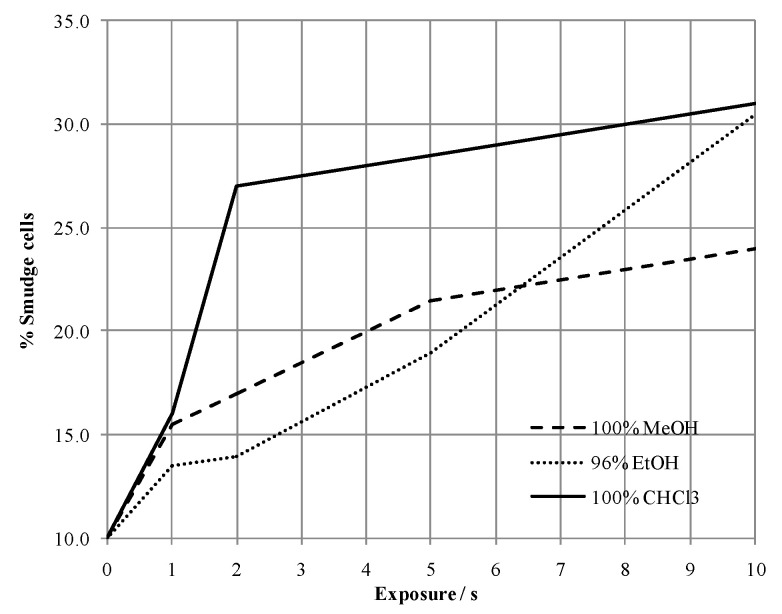
White blood cell (WBC) destruction due to exposure to solvents producing so-called “smudge cells”.

Removal of the blood plasma that covers cells of interest was performed using polar solvents. This procedure causes, among other things, WBC destruction, leaving cell remnants called smudge cells, the count of which served as the sample integrity quality indicator (Figure 2). As a compromise between the cell destruction and removal of interfering plasma components, 5 s of exposure to 96% EtOH was selected for the following MSI experiments.

The selection of 9–aminoacridine (9AA) and α-cyano-4-hydroxycinnamic acid (CHCA) deposition conditions, LASER intensities, and pitch sizes for D0 and D1 instrumental settings was performed using heme, ATP, and IMP signal intensities as quality indicators: the results are given in the Supplementary Materials (Figures S1–S5). Five-minute sublimation of 9AA or CHCA followed by 105 s of recrystallization was selected for the following experiments. The method development produced the following instrumental settings:

1. D0: 80 nJ LASER energy (LASER intensity of 12% and 100 Hz frequency), x and y pitch sizes of 8 and 7 µm, and 50 LASER shots per pixel were predefined;
2. D1: 95 nJ LASER energy (LASER intensity of 14% and 40 Hz frequency), x and y pitch sizes of 8 and 6 µm, and 30 LASER shots per pixel were predefined.

**Figure 3:**
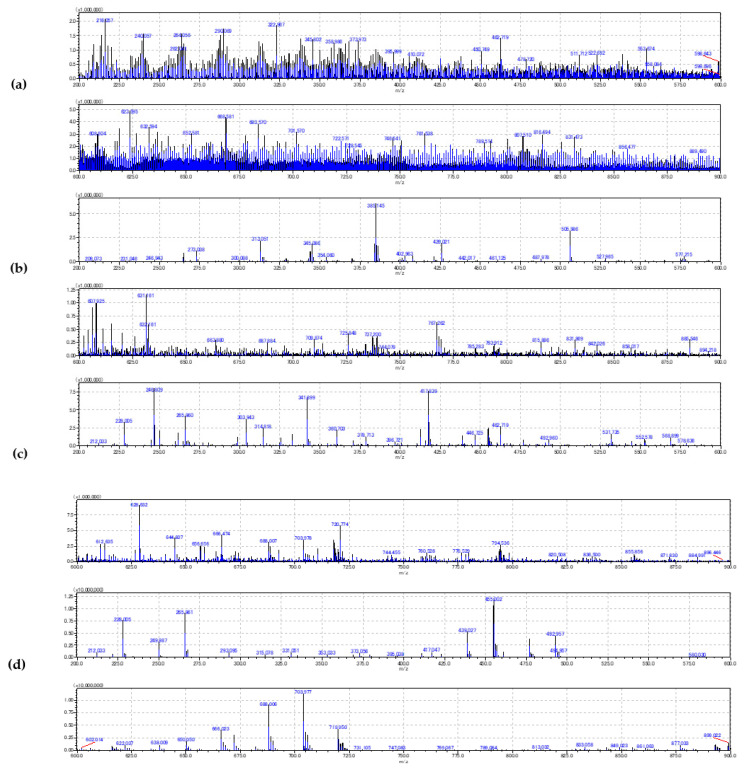
Comparison of total ion count (TIC) normalized spectra (upper: *m*/*z* 200–600 and lower: 600–900 Da) averaged over all pixels recorded using the selected D0 and D1 instrumental settings: (**a**) D0 settings and 9–aminoacridine (9AA) matrix/negative ionization, (**b**) D1 settings and 9AA matrix/negative ionization, (**c**) D0 settings and α-cyano-4-hydroxycinnamic acid (CHCA) matrix/positive ionization, and (**d**) D1 settings and CHCA matrix/positive ionization.

During method development, the focus was on the *m*/*z* signal intensities of cellular markers. Complete total ion count (TIC)-normalized spectra recorded using the selected instrumental conditions are given in Figure 3.

**Figure 4:**
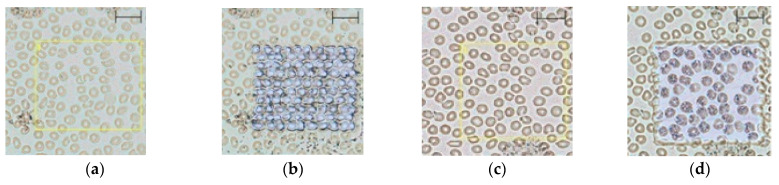
LASER footprint: (**a**) light microscopy image of a blood smear covered by 9AA, (**b**) the same optical field as in 4a after MSI was performed using D0, (**c**) light microscopy image of a blood smear covered by 9AA, and (**d**) the same optical field as in 4c after MSI was performed using D1. Optimized sample preparation and instrumental conditions were used. Scale bar corresponds to 20 µm.

LASER shot diameter and accuracy are of critical importance for the reliable generation of MSI and individual cell chemical composition assessment. Figure 4 shows regions of interest (ROI) designated by yellow squares before and after the MSI was performed using the selected D0 and D1 settings.

Cells surrounding the yellow square in Figure 4a show that the same square was shot by LASER in Figure 4b. For example, it can be noticed that the RBC, which lies in the upper left corner of the square in Figure 4a, has been shot by the LASER and that the corresponding LASER footprint covers the same cell in Figure 4b. The LASER footprint visible in Figure 4b corresponds to the LASER-caused matrix changes not cellular changes.

### 2.2. Evaluation of the Blood Smear MSI

**Figure 5:**
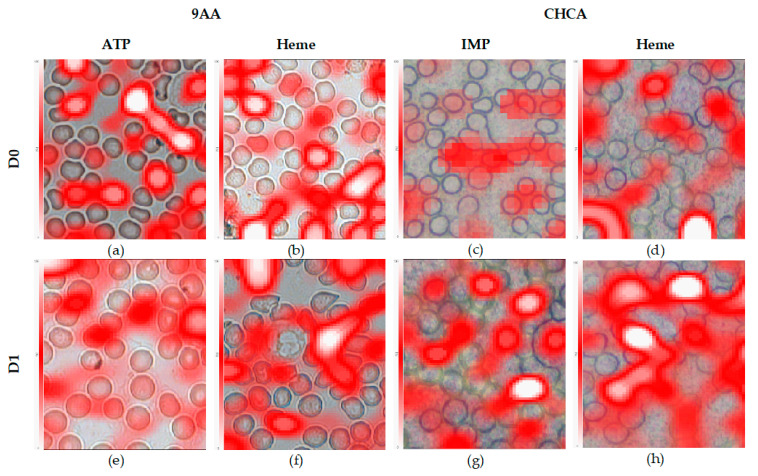
Lateral distribution of cellular markers overlaid on light microscopy images: ATP (**a**,**e**), heme (**b**,**d**,**f**,**h**), and inosine monophosphate (IMP) (**c**,**g**). MSI (**a**–**d**) was recorded using the selected D0, while images (**e**–**h**) were recorded using selected D1 settings. The 9AA matrix and negative ionization were used for recording images (**a**,**b**,**e**,**f**). The CHCA matrix and positive ionization were used for recording images (**c**,**d**,**g**,**h**). Scale bar corresponds to 5 μm.

Quality of MSI represents the ultimate goal of the method development: examples of images recorded using selected sample preparation and instrumental settings are given in Figure 5.

Differential *m*/*z* signal expression analysis relied on a comparison of the RBC and WBC signals with signals coming from the plasma remnants: the statistically significant results of the two sample *t*-test using samples coming from 6 different blood donors are given in Table 1 and Table 2. Table 1 contains counts of the differentially expressed *m*/*z* signals that were recorded using different instrumental settings and different matrices (Figure 3a–d). Table 2 contains metabolites identified in the Human Metabolome Database (HMDB): only the single database hits having a Kyoto Encyclopedia of Genes and Genomes (KEGG) identifier which belongs to the group of endogenous or exogenous but essential compounds are presented, i.e., multiple database search hits, including isobaric ions, were excluded from the following analysis. The results given in Table 2 were integrated over all MSI settings tested.

## 3. Discussion

### 3.1. Sample Preparation and Instrumental Settings

**Figure 6:**
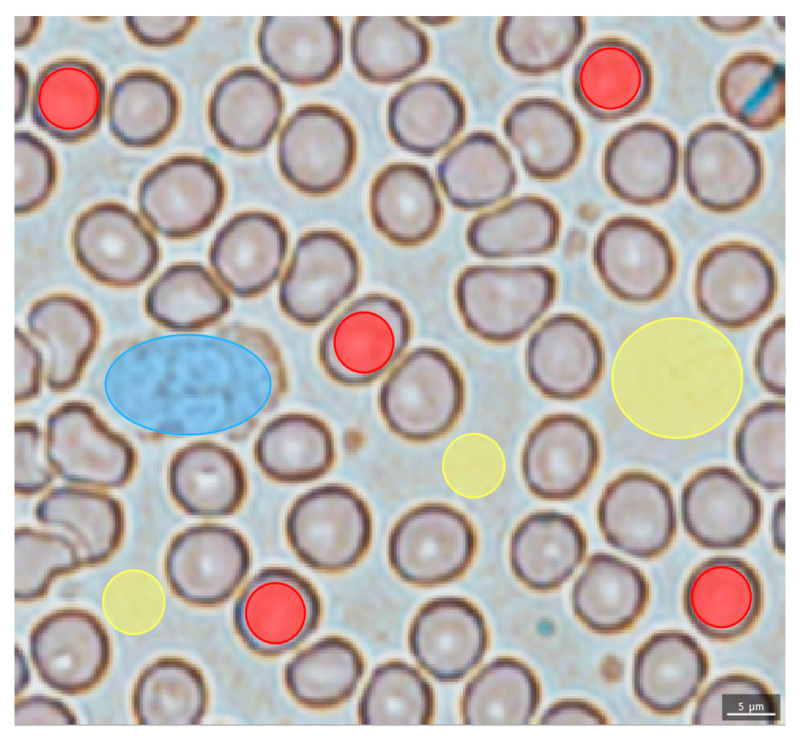
Regions of interest (ROI) selection: red ellipses cover RBCs, light blue ellipse covers WBCs, and yellow ellipses cover plasma remnant regions. Light microscopy image: blood smear covered by a 9AA matrix.

Blood smeared over the ITO slide forms a single cell layer that is firmly attached to the surface even without a fixation agent (Figure 4, Figure 5 and Figure 6). Zones of different cell dispersions/densities are clearly visible (Figure 6). Among those, the zone containing the lowest surface density of blood cells that had not been clumped together was chosen for MSI. The 9AA and CHCA matrices deposited by sublimation lasting 5 min formed transparent layers over the smear (Figure 4 and Figure 5), but at least in the D0 case, *m*/*z* signals recorded using a negative ionization were relatively weak, which may be attributed to the low LASER energies and thin matrix layer (Figure 3). The noisy appearance of spectra recorded using negative ionization/9AA matrix (Figure 3a,b) may be associated with easily ionized free fatty acids (FFA) and phospholipids: both blood plasma and different blood cells contain significant quantities of these compounds [4,14,19]. Even in the best-case scenarios provided by short exposure to EtOH and MeOH, there was a significant trade-off between the sample integrity and plasma removal (Figure 1): careful visual inspection of Figure 4 confirms the existence of plasma remnants in regions surrounding blood cells. All blood cells, while circulating in vascular system, are immersed in the plasma: plasma removal using more effective agents not only could cause profuse cell destruction but also could compromise the assessment of blood cell chemical composition. However, stronger heme and ATP *m*/*z* signals recorded in negative ionization/9AA mode using selected D1 in comparison to D0 settings suggest that oversampling may explain the noisy appearance of the spectra: smaller LASER spot sizes may leave blood cells intact, leading to domination of easily ionized plasma lipids covering the cell surfaces (Figure 3a vs. Figure 3b and Figure 5a,b vs. Figure 5e,f, Supplementary Figures S3–S5) [18]. The mass spectra given in Figure 3a were recorded using the same slide as in Figure 3b. A lower LASER energy in the D0 case is the most probable cause of different appearances in these figures. A D0 vs. D1 comparison using positive ionization/CHCA matrix, heme and IMP as cellular markers shows the same trend: weak intracellular marker signals are associated with nonoverlapping low energy LASER spots, i.e., D0 settings, while application of oversampling, i.e., D1 settings, produces stronger *m*/*z* signals probably due to the increased cell wall permeability and/or more desorbed material (Figure 3c vs. Figure 3d and Figure 5c,d vs. Figure 5g,h, Supplementary Figures S2, S4, and S5).

Evaluation of the correspondence between the light microscopy image and LASER footprint preceded the MSI experiments. As shown in Figure 4, total LASER footprints recorded using the selected D0 or D1 settings correspond well with the preselected ROI. Graphical representation of the following MSI experiments is given in Figure 5. As expected, the strongest signal intensities coming from the cellular markers are located over or in close vicinity to the cells while the non-populated spaces correspond to the weaker signals. Lateral distribution of the cellular markers is consistent with the cell type specificity: while heme-associated *m*/*z* signals cover primarily RBC, the ATP-associated *m*/*z* signals are shared by WBC and RBC [1]. IMP signals are too weak to be analyzed in detail, but even these may be detected over some RBC, as expected [3]. Individual cells are not expected to have the same content of metabolites. Therefore, the different *m*/*z* signal intensities associated with individual cells is an expected outcome. However, some signals are “smeared” over the spaces surrounding cells: in the D1 case, this can be attributed to LASER spot overlap and to the leakage of cellular components as a result of the EtOH treatment or cell death. LASER spot overlap raises the possibility of mixing the mass spectra coming from pixels of interest with the spectra of its surroundings (Figure 4). Besides, in some instances, the LASER spot center does not perfectly match the center of some particular cell. On the other hand, in the D1 case, most cells are covered by chosen *m*/*z* signals, but in the D0 case, chosen *m*/*z* signals cover a smaller fraction of existing cells (Figure 5). Partial coverage of blood cells with *m*/*z* signals coming from the cellular markers in the D0 case probably does not provide the true cellular markers’ content: it is rather an artefact of weak desorption or ionization.

Application of the *t*-test to MSI sets produced differentially expressed *m*/*z* for each cell type, each matrix, and each instrumental setting evaluated (Table 1): RBC and D1 settings produced fewer differentially expressed *m*/*z* in comparison to WBC and D0 settings, respectively, while the CHCA matrix enabled detection of more differentially expressed *m*/*z* then the 9AA matrix. The first trend is supposed to be associated with the fact that RBCs have less complex metabolic networks and more resistant membranes. This result may also mean that RBCs show greater cell-to-cell variability: 5 RBCs vs. only 1 WBC per blood donor were included in differential *m*/*z* expression analysis. The second trend is supposed to be associated with mixing of the neighboring pixel’s spectra: in the case of D0 experiments, which did not cause LASER spot overlap, neighboring LASER spot spectra mixing is less probable (Figure 4). However, due to the stronger signals, only the application of D1 settings in positive ionization mode produced significant differences in the heme-associated *m*/*z* signal between the RBC and plasma. The third trend reflects the greater fraction of neutral and basic metabolites that are prone to positive ionization and the somewhat stronger signals coming from CHCA-treated samples.

### 3.2. Human Metabolome Database Search

Differential expression analysis of the *m*/*z* signals coming from the RBC, WBC, or blood plasma was performed using *t*-test. The cells were compared to plasma, and significantly different *m*/*z* signals entered the HMDB and KEGG search using ±0.025 Da tolerance. Single hits coming from this search that correspond to endogenous or essential compounds were integrated over all evaluated chemical and instrumental settings, while multiple search hits, including isobaric compounds, were eliminated. In comparison to the total count of differentially expressed *m*/*z* signals given in Table 1, the total count of differentially expressed metabolites given in Table 2 is substantially lower due to the excluded multiple database search hits and due to the *m*/*z* values that did not correspond to any of existing database entries.

The results presented in Table 2 corroborate the association of differentially expressed metabolites with appropriate blood cell types. Among the identified metabolites, the ones in which cellular content decreased in comparison to plasma predominated. RBC and WBC contents of lipids like acetyl-CoA are seemingly decreased, but this primarily reflects the fact that blood plasma represents the major medium for their transport. The same stands for some hormone metabolites like 3,5-diiodo-l-tyrosine and small peptides like *N*-acetylaspartylglutamic acid. The energetic demands of cells, especially WBC, are reflected in the decreased content of NADPH, NADHX, etc. The number of metabolites for which content is increased in cells in comparison to plasma is not too many, but these metabolites may provide more convincing evidence of blood smear MSI performance. Contents of dATP were increased in both cell types in comparison to plasma. In WBC, dATP is a substrate and inhibitor of DNA synthesis, and in RBC, it may serve as a secondary source of energy in case of ATP depletion [20]. This metabolite inhibits DNA synthesis in WBC by inhibition of ribonucleotide reductase, but it also inhibits *S*-adenosylhomocysteine (SAH) hydrolase in both cell types. This in turn leads to depletion of deoxynucleotides like dCDP in WBC and to accumulation of demethylated metabolites like l-homocysteic acid accompanied by depletion of methylated products like 5-methyldihydrofolic acid and (6R)-5,10-methenyltetrahydrofolate detected in RBC and WBC, respectively. Among the metabolites which content is increased in both cell types, l-glutamic acid 5-phosphate stands out: it is required for the removal of ammonia from the cells, and as a precursor for the glutathione synthesis, it is essential for the proper response to oxidative stress. Therefore, it is expected that l-glutamic acid 5-phosphate’s content is increased in both cell types in comparison to plasma. On the other hand, the accumulation of heme and thiamine diphosphate is specific to RBC: heme plays a crucial role in RBC-mediated oxygen transport, while thiamine pyrophosphate plays a crucial role in the RBC-specific pentose phosphate pathway. Dihydroneopterin phosphate accumulates in WBC as expected due to its role in WBC-mediated inflammatory response [21]. Selenoproteins and molybdate listed in Table 2 are endogenous compounds physiologically present in the blood cells, and their roll in cell metabolism is described in the literature [22,23,24]. The association of the rest of the metabolites (Table 2) with different cell types is omitted since they are either redundant or there is a lack of evidence of their cell-specific role and expression.

## 4. Materials and Methods

### 4.1. Human Subjects

This study was conducted at the Osijek University Hospital, Croatia from March to July 2020 in accordance with the Declaration of Helsinki. The study was approved by the hospital’s ethical committee. Fifty-eight subjects were enrolled in the study. All subjects were apparently healthy male blood donors aged 20–60 who provided written consent for participation in the study.

### 4.2. Sample Preparation

Approximately 3 mL of blood from the antecubital vein of each subject was collected in the sample tube containing EDTA anticoagulant (Becton Dickinson, Plymouth, UK). Sample suitability evaluation that involved RBC and WBC counts was performed using the XN-2000 hematology analyzer (Sysmex, Kobe, Japan): blood samples characterized by the WBC or RBC counts beyond the reference ranges were excluded from the study [1]. Unless stated otherwise, 2 µL of each blood sample was smeared over the unused ITO slide of 15–25 Ω/sq surface resistivity (Sigma Aldrich, St. Louis, MO, USA). The smeared blood was left to dry on the ITO slide for 5 min under room conditions. Two blood smears per each ITO slide were prepared and analyzed within the 24 h period after the venipuncture took place.

ITO slides with blood smears were immersed in methanol Ph. Eur. (MeOH; Merck, Darmstadt, Germany), chloroform p.a. (CHCl_3_; Kemika, Zagreb, Croatia), or 96% EtOH Ph. Eur. (Merck, Darmstadt, Germany) for 1, 2, 5, and 10 s. The percentage of disintegrated WBC called “smudge cells” associated with exposure to the solvents was used only as the sample integrity indicator (these cells were not analyzed). Due to favorable sublimation properties and transparent appearance, 9AA (Sigma Aldrich, St. Louis, MO, USA) and CHCA (Sigma Aldrich, St. Louis, MO, USA) matrices were chosen for the study. Three-, five-, and eight-min lasting matrix sublimation procedures were evaluated using iMLayer (Shimadzu, Kyoto, Japan) in accordance with the manufacturer’s settings. Recrystallization was performed in a closed container heated to 37 °C for 300 s or 70 °C for 105 s using 5% *v*/*v* MeOH:H_2_O solution: blood-smeared ITO slides covered with the matrix were placed above the solution. Indicators of the matrix deposition quality were the visibility of matrix-covered WBC and TIC-normalized signal intensities of cellular markers like heme, ATP, and IMP averaged over all pixels. The corresponding *m*/*z* values are given in Table 3. A tolerance of ±0.025 Da was used for the detection of corresponding signals in the recorded mass spectra.

### 4.3. Instrumental Settings

All MSI experiments were performed using iMScope Trio (Shimadzu, Kyoto, Japan). The instrument was equipped with a light microscope, Nd:YAG LASER (355 nm), containing an atmospheric pressure MALDI source and an ion trap (IT) TOF MS running in the reflectron mode. The light microscopy settings used throughout the study included non-stained samples, 400× magnification, and an incident or transmitted light source. The critical parameters for instrumental settings selection were LASER spot size and total LASER footprint, which should conform to the analysis of individual blood cells dispersed over the ITO surface. Different instrumental settings were examined (Supplementary Figures S2–S5), among which two experimental settings which produced the best results were selected for the following experiments: the first set was associated with the nominal LASER diameter (named D0) of 5 μm and the second one (named D1) was associated with the nominal LASER diameter of 10 µm. To accomplish better MS sensitivity while keeping the LASER spot small, narrow *m*/*z* ranges were coupled to low LASER frequency and a low number of LASER shots per pixel. If not stated otherwise, each MSI experiment involved recording mass spectra across two neighboring *m*/*z* ranges: 200–600 and 600–900 Da using negative and positive atmospheric MALDI ionization and maximum source voltage. Four MS images per MSI experiment using D0 settings were recorded, and they contained ca. 100 pixels. The same stands for the D1 settings. Some settings were fixed throughout the study: D0 settings always involved 50 spectra per pixel coupled with 100 Hz LASER frequency, while D1 settings always involved 30 spectra per pixel coupled with 40 Hz LASER frequency. On the other hand, different LASER energies and pitch sizes were tested during method development. LASER energies ranging from 69 to 130 nJ together with x and y pixel pitch sizes ranging from 6.0 and 5.0 to 8.0 and 7.0 µm were evaluated as a part of D0 setting selection, while energies ranging from 95 to 194 nJ together with x and y pixel pitch sizes ranging from 8.0 and 6.0 to 14.0 and 12.0 µm were evaluated as a part of D1 setting selection. *m*/*z* signal intensities coming from the cellular markers were used as quality indicators. All MSI experiments involved two blood smears coming from two different donors. After the method development was finished, the selected MSI conditions were applied to the analysis of differentially expressed *m*/*z*. A new set of blood smears was used for that purpose.

### 4.4. Data Analysis

Imaging MS Solution 1.30.06 (Shimadzu, Kyoto, Japan) was used for the average mass spectrum visual inspection. All MSI were generated by IMAGEREVEAL 1.1 (Shimadzu, Kyoto, Japan). Lanczos interpolation, median smoothing, and ±0.025 Da tolerance were used for that purpose. Targeted analysis was followed by a differential analysis. Targeted analysis was performed using TIC-normalized centroid signals found in 0.020 Da *m*/*z* bins, for which the intensity was greater than 0.1% of the highest signal in the mass spectrum. Five RBCs, one WBC, and three plasma remnant ROIs per smear coming from six different individuals were selected for the collection of RBC-, WBC-, and plasma-specific mass spectra (Figure 6).

This procedure was applied on 8 different sets of MSI that were recorded using D0 or D1 LASER settings, two different *m*/*z* ranges, and positive or negative ionization. Recorded spectra were preprocessed as described earlier in the text, but only 500 most intense *m*/*z* per single MSI set were kept for the differential expression analysis. *t*-test and 0.05 significance limit were used for the comparison of the RBC spectra or WBC spectra with the spectra coming from the seemingly empty areas of each smear that actually contained some blood plasma remnants. The obtained differentially expressed *m*/*z* were used as inputs for the HMDB search using ±0.025 Da tolerance [25]: unique HMDB hits associated with the endogenous and exogenous essential compounds having KEGG ID were used for the differential analysis [26].

## 5. Conclusions

Sample preparation and instrumental settings suitable for the MALDI TOF MSI of blood smear were developed. The sample preparation consisted of transparent matrix deposition by sublimation onto ITO slides containing blood smears briefly treated with EtOH. Two sets of instrumental MALDI TOF MSI conditions were evaluated using cellular markers and differential expression analysis. MSI coincided well with the light microscopy images recorded using integrated light microscope and MALDI TOF MS. Lateral distributions of cellular markers were consistent with the respective cell type marker’s content. Differential analysis performed on RBC and WBC followed by the metabolite database search produced hits that were consistent with the respective cell types. In conclusion, this study sets the foundations for application of blood smear MSI in clinical diagnostics and research.

## Figures and Tables

**Table 1:** Differentially expressed *m*/*z* signal counts.

Instrumental Settings	D0			D1	
Cell Type	*m*/*z*	9AA	CHCA	9AA	CHCA
RBC	200–900 ^a^	60	100	29	82
WBC	200–900 ^a^	68	145	80	100

^a^ Results coming from two consecutive *m*/*z* ranges were combined.

**Table 2:** Metabolites differentially expressed in RBC and WBC relative to plasma: metabolites were assigned to appropriate *m*/*z* signals by Human Metabolome Database (HMDB) and Kyoto Encyclopedia of Genes and Genomes (KEGG) database searches using ±0.025 Da tolerance. Multiple database search hits like isobars were excluded: only the single hits that belong to endogenous or exogenous but essential compounds are given.

*m*/*z*	Adduct	KEGG ID	Metabolite	Cell/Plasma	Instrumental Settings	Cell Type
367.99194	M-H20-H	C00705	2′-Deoxycytidine diphosphate (dCDP)	0.8	D0−	RBC
423.02167	M-H	C00068	Thiamin diphosphate	>0.1	D1−	RBC
607.17777	2M-H	C12270	*N*-Acetylaspartylglutamic acid	0.3		
743.53308	2M-H	C13828	Cervonoyl ethanolamide	6.5		
755.09679	M-H	C06197	Diadenosine triphosphate	>0.1		
821.05335	2M-H	C00206	2′-Deoxyadenosine 5′-diphosphate	>0.1		
221.97144	M+K	C16511	l-Homocysteic acid	1.6	D0+	RBC
265.96063	M+K	C03287	l-Glutamic acid 5-phosphate	1.2		
326.77131	2M+H	C06232	Molybdate	2.0		
415.84174	M+H-H_2_O	C01060	3,5-Diiodo-l-tyrosine	0.8		
456.0047	M+H-2H_2_O	C00131	2′-Deoxyadenosine 5′-triphosphate (dATP)	1.4		
500.04952	M+Na	C00513	CDP-glycerol	2.8		
514.96755	M+Na	C01345	2′-Deoxyinosine triphosphate	0.5		
694.83621	2M+Na	C05704	Selenocystine	0.7		
790.43199	M+K	C06125	3-O-Sulfogalactosylceramide (d18:1/14:0)	0.7		
418.04554	M+K	C03451	*S*-Lactoylglutathione	0.6	D1+	RBC
498.14504	M+K	C00440	5-Methyltetrahydrofolic acid	1.2		
616.17073	M+H	C00032	Heme	1.6		
652.04788	M+H-2H_2_O	C04426	Uridine diphosphate acetylgalactosamine 4-sulfate	0.7		
774.11178	M+H-2H_2_O	C00024	Acetyl-CoA	0.6		
796.1011	M+H	C00798	Formyl-CoA	0.9		
338.96305	2M-H	C11499	(*S*)-3-Sulfonatolactate	0.5	D0−	WBC
367.99194	M-H20-H	C00705	dCDP	0.6		
409.06403	2M-H	C02470	Xanthurenic acid	1.7		
601.60302	2M-H	C00836	Sphinganine	0.6		
409.00689	M-H20-H	C00104	Inosine 5′-diphosphate (IDP)	0.8	D1−	WBC
426.95079	2M-H	C06054	2-Oxo-3-hydroxy-4-phosphobutanoic acid	17.8		
488.97877	M-H20-H	C00081	Inosine triphosphate (ITP)	3.2		
506.11841	M+Cl	C03204	10-Formyldihydrofolate	9.2		
506.99341	M-H	C00081	ITP	1.9		
724.08168	M-H20-H	C00006	NADP	>0.1		
743.53308	2M-H	C13828	Cervonoyl ethanolamide	0.3		
753.97899	M+Cl	C02739	Phosphoribosyl-ATP	>0.1		
806.70364	M-H20-H	C01190	GlcCer(d18:1/25:0)	>0.1		
221.97144	M+K	C16511	l-Homocysteic acid	1.8	D0+	WBC
265.96063	M+K	C03287	l-Glutamic acid 5-phosphate	1.5		
326.9293	2M+Na	C05527	3-Sulfinylpyruvic acid	0.5		
376.89968	2M+K	C05688	l-Selenocysteine	2.8		
415.84174	M+H-H_2_O	C01060	3,5-Diiodo-l-tyrosine	0.7		
456.0047	M+H-2H_2_O	C00131	Deoxyadenosine triphosphate	1.8		
694.83621	2M+Na	C05704	Selenocystine	0.6		
701.98995	M+H-H_2_O	C02739	Phosphoribosyl-ATP	0.6		
709.07265	2M+K	C05925	Dihydroneopterin phosphate	1.7		
762.92406	2M+H-H_2_O	C00119	Phosphoribosyl pyrophosphate	0.6		
478.16774	M+Na	C00445	(6R)-5,10-methenyltetrahydrofolate	0.7	D1+	WBC
586.02355	M+H-2H_2_O	C19851	ADP-ribose 1″-2″ cyclic phosphate	0.4		
706.13261	M+Na	C04856	NADHX	0.4		
728.10302	M+H-H_2_O	C00005	NADPH	0.9		
779.09063	M+Na	C06197	Diadenosine triphosphate	1.0		
865.13597	2M+H	C05692	Se-Adenosyl-l-selenohomocysteine	0.5		

**Table 3:** Cellular marker compounds used as the MSI quality indicators.

Ionization	Cellular Marker Compound	Adduct Ion	Monoisotopic *m*/*z* (Da)
Negative	ATP	[M − H]^−^	505.988
	Heme (Fe II)	[M − H]^−^	615.170
Positive	IMP	[M + Na]^+^	371.037
	Heme (Fe III)	[M]^+^	616.177

## Data Availability

The raw data is available on request that should be send to: zeljko.debeljak@gmail.com.

## Supplementary Materials

Supplementary materials can be found at https://www.mdpi.com/1422-0067/22/2/585/s1.

## Abbreviations

9AA	9-aminoacridine
ATP	Adenosine triphosphate
CHCA	A-cyano-4-hydroxycinnamic acid
EDTA	Ethylenediaminetetraacetic acid
HMDB	Human metabolome database
IMP	Inosine monophosphate
IT	Ion trap
ITO	Indium tin oxide
KEGG	Kyoto Encyclopedia of Genes and Genomes
MALDI	Matrix assisted LASER desorption ionization
LC-MS/MS	Liquid chromatography coupled to tandem mass spectrometry
MSI	Mass spectrometry imaging
RBC	Red blood cells
ROI	Region of interest
SIMS	Secondary ion mass spectrometry
TIC	Total ion count
TOF	Time-of-flight
WBC	White blood cells
